# Crystal Morphology Prediction and Anisotropic Evolution of 1,1-Diamino-2,2-dinitroethylene (FOX-7) by Temperature Tuning

**DOI:** 10.1038/s41598-020-59261-3

**Published:** 2020-02-11

**Authors:** Liang Song, Feng-Qi Zhao, Si-Yu Xu, Xue-Hai Ju, Cai-Chao Ye

**Affiliations:** 1 Key Laboratory of Soft Chemistry and Functional Materials of MOE, School of Chemical Engineering, Nanjing University of Science and Technology, Nanjing, 210094 China; 2 Science and Technology on Combustion and Explosion Laboratory, Xi’an Modern Chemistry Research Institute, Xi’an, 710065 China; 3 Academy for Advanced Interdisciplinary Studies & Guangdong Provincial Key Laboratory of Computational Science and Material Design, Southern University of Science and Technology, Shenzhen, 518055 China

**Keywords:** Computational chemistry, Molecular dynamics

## Abstract

Temperature-induced morphological changes are one of the strategies for designing crystal shapes, but the role of temperature in enhancing or inhibiting crystal growth is not well understood yet. To meet the requirements of high density and low sensitivity, we need to control the crystal morphology of the energetic materials. We studied the crystal morphology of 1,1-diamino-2,2-dinitroethylene (FOX-7) in dimethyl sulfoxide/water mixed solvent by using the modified Hartman-Perdok theorem. Molecular dynamics simulations were used to determine the interaction of FOX-7 and solvents. The results showed that the crystal shape of FOX-7 is hexagonal, the (101) face is the largest exposed face and is adjacent to six crystal faces at 354 K. As the temperature goes down, the area of the (001) face is significantly reduced. The crystal morphology of FOX-7 at 324 K has a smaller aspect ratio of 4.72, and this temperature is suitable for tuning the morphology from slender hexagon into diamond. The prediction results are in remarkable agreement with the experiments. Moreover, we predicted the evolution path of FOX-7 morphology by Gibbs-Curie-Wulff theorem and explained the variation of crystal shape caused by different external conditions in the actual crystallization process.

## Introduction

Crystallization as a green separation process is characterized by low energy consumption and high efficiency^1–3^. The growth shape that a crystal obtains in the course of its formation is also highly sensitive to growth conditions, and reflects the growth mechanism, as does the surface morphology^4–7^. Therefore, the growth shapes make it possible to judge the formation conditions and to correct the growth parameters in experiment. Based on the periodic bond chain (PBC) theory, Hartman and Perdok stated that the growth rate of a face is lower if fewer chains of strong bonds cross this face^8,9^. This is the well-known Hartman-Perdok theorem (H-P theorem). The equilibrium crystal morphology can be determined by calculating the attachment energy. Gibbs stated that the polyhedral features of the crystal shape reduce the total surface energy. Curie defined that the normal growth rate of the crystal face is in proportion to the surface free energy^10^. Wulff further proved that the distance from the crystal center to crystal face is proportional to the specific surface free energy in equilibrium^11^. This relationship is also called the Gibbs-Curie-Wulff theorem^12,13^. The prediction and regulation of crystal morphology is essential in the fields of pharmaceuticals^14,15^, catalysis^16,17^, functional ceramics^18^, thin film materials^19^, energetic materials^20,21^, etc. For example, the crystal morphologies influence the sensitivity of the energetic materials. In the formation of a drug product, crystals with high aspect ratio shapes are troublesome in the subsequent processing steps. In the field of catalysis, it is efficient for the catalyst to expose more active crystal face. Anisotropic morphologies, especially the large aspect ratio of one-dimensional needle/line and two-dimensional sheet, have received wide attention. The external environment regulates the anisotropic growth of crystals^22–25^. The use of additives has proven to be an effective way to modulate anisotropic growth morphology. In addition, quantitative reaction or mass transfer control during crystallization can also effectively adjust surface bonding behavior, thereby control the growth rate of different crystal faces. For materials with isotropic crystal structures, the selective formation of precursors with the desired anisotropic morphology has become the primary design tool.

We need to control the crystal morphology of energetic materials in order to satisfy high bulk density and to reduce sensitivity^26,27^. According to the generally accepted “hot spot” theory, it is believed that the explosive detonation is divided into the hot spot formation stage and the hot spot growth into the explosion stage^28,29^. Under the external mechanical force, the total probability of the explosive reaction generating explosion depends on the hot spot generation and propagation. The formation of the hot spot is mainly determined by the physical and mechanical properties of the explosive crystal or particle. Crystal morphologies having a large aspect ratio such as needle and rod shape are likely to form hot spots. Liu *et al*. confirmed that the crystal morphologies have a significant impact on the sensitivity by culturing the plate and rod crystals of 3,4-bis(3-nitrofurazan-4-yl)furoxan (DNTF) grown in water/acetic acid and water/ethanol solvents, respectively^30^. Similar results were observed for the morphologies of 2,4,6,8,10,12-hexanitro-2,4,6,8,10,12-hexaazaisowurtzitane (CL-20) and cocrystals of CL-20 and hexahydro-1,3,5-trinitro-1,3,5-triazine (RDX)^31^. Improving the crystal quality of the explosive (controlling the crystal morphology, eliminating crystal defects, etc.) and coating the explosive crystals (coated by polymers, surfactants and insensitive agents, etc.) can reduce the probability of hot spots. Therefore, the recrystallization can improve the crystal morphology of the elemental explosive and reduce the crystal defects. Here, 1,1-diamino-2,2-dinitroethylene (FOX-7) was selected as a candidate because it is a new type of high-energy insensitive explosive, and has excellent performance and practicality^32,33^. It has specific functional groups (nitro and amino groups), thus it can form strong hydrogen bonds and dipole interactions. Consequently, the binding force of homogeneous liquids on its crystal face growth is greater. Zhao *et al*. used a modified attachment energy model to study the effect of different solvents on the morphology of FOX-7 crystals^34^. Shim *et al*. found that the aspect ratio of FOX-7 crystal decreases as the cooling rate of temperature increases in dimethylacetamide/water solvent^35^. They predicted and explained this phenomenon by step energy calculation and kinetic Monte Carlo simulation. Li *et al*. introduced the Gibbs-Curie-Wulff theorem into the field of organic materials, and explained the growth morphology of dibenzo[d,d′]thieno[3,2-b;4,5-b′]dithiophene in vapor phase conditions by calculating the relationship between surface energy and crystal growth^36^. In this paper, we quantified the thermodynamic ideal crystal shape of FOX-7 by the H-P theorem. The anisotropy of the microstructure is fully reflected in the macroscopic shape by the ideal form of thermodynamics. Taking into account the external environment, the evolution of anisotropic growth morphology under additive and temperature control was simulated by the interface model of actual crystal growth, and the key role of adsorption behavior was revealed in the formation of anisotropic growth morphology.

## Results and Discussion

**Figure 1:**
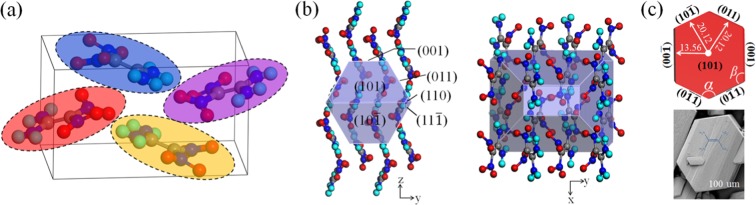
(**a**) Unit cell of FOX-7, (**b**) Predicted crystal shape of FOX-7 using the H-P method in vacuum, (**c**) Predicted and experimental shape of FOX-7 crystal in DMSO/H_2_O = 2:1 and at 354 K. Embedded graph in the lower right corner is the experimental shape^37^.

As a typical insensitive energetic material, the crystal morphology of FOX-7 has an important influence on safety performance and formulation filling method. How to effectively adjust its anisotropic growth morphology is one of the important ways to obtain excellent physical/chemical properties. Reasonable use of additives has proven to be an effective way to increase or decrease the anisotropic growth rate of FOX-7 in different crystallographic directions. Figure 1(a) shows the crystal structure and molecular symmetry position. The FOX-7 unit cell belongs to the monoclinic space group of *P2*_1_*/n*. Four FOX-7 molecules fill a primitive unit cell structure. One carbon atom of FOX-7 molecule connects to two amino groups, and another connects to two nitro groups, forming an electron push-pull ethylene structure. It belongs to the asymmetric central molecule, which increases the influence of every crystal face on the growth environment. There are inherent differences in crystal structure, such as the difference in lattice arrangement in different directions, the influence of the rotating axis and the slip surface. The crystal shape of FOX-7 in vacuum estimated by using the H-P method is shown in Fig. 1(b). On this basis, further attempts were made to treat the solvent effect on the acquired crystal face. A scenario with a temperature of 354 K in dimethyl sulfoxide/water (DMSO/H_2_O) = 2:1 was used. The crystal shape shown in Fig. 1(c) exhibits a distinct hexagon and this is consistent with the experimental results^37^. The growth crystal orientation of FOX-7 is such that its (101) face is parallel to the substrate, and the largest face of the hexagonal crystal is (101). Furthermore, according to Steno’s law (the constant law of the interface angle) and the regular hexagon of the crystal, the facet angles of 120.22° (*α*) and 119.89° (*β*) are predicted between (0$\bar{1}$1) face and its two neighbor faces (Fig. 1c). The parameters of the crystal faces of the FOX-7 are shown in Table 1. Obviously, in the growth environment, the surface adsorption energy of (101) is the lowest, that is, the growth rate is the slowest, and the exposed surface area is the largest. The adsorption energies of (001) and (011) faces are smaller than that of (101) face by 74.42% and 80.22%, respectively, indicating that the growth rates in these directions are significantly larger than (101) face. The adsorption energies of (10$\bar{1}$), (110) and (11$\bar{1}$) are larger than those of the above surfaces, and these faces should not appear in the Wulff structure.

**Table 1:** Interplanar distance (*d*_hkl_), roughness (*R*) and adsorption energy (*E*) of FOX-7^a^.

(*hkl*)	*d*_hkl_	*R*(%)	*E*_H–P_^b^	${{\boldsymbol{E}}}_{{\bf{H}}{\boldsymbol{-}}{\bf{p}}}^{{\bf{Mod}}\,{\bf{c}}}$	R_*i*_/*R*_101_^b^	*R*_*i*_^*Mod*^/*R*_101_^c^
(101)	5.97	1.38	−30.67	−3.98	1.00	1.00
(10$\overline{1}$)	5.82	1.30	−30.40	−14.07	0.99	3.54
(001)	5.70	1.69	−41.35	−15.56	1.35	3.91
(011)	5.69	1.15	−37.47	−20.12	1.22	5.06
(110)	4.75	1.58	−51.04	−25.14	1.66	6.32
(11$\overline{1}$)	4.35	1.20	−46.67	−26.07	1.52	6.55

^a^All energies are in kcal mol^−1^, distances are in Å. ^b^In vacuum. ^c^In DMSO/H_2_O = 2:1 (354 K).

**Figure 2:**
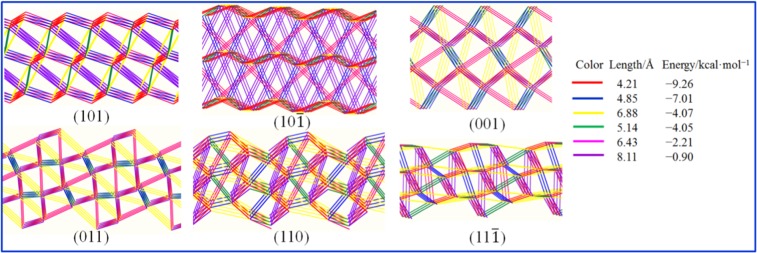
Three-dimensional bonding networks for the (101), (10 $\bar{1}$), (001), (011), (110) and (11 $\bar{1}$) faces. Length is the distance between the centers of the two molecules, and energy is the interaction energy between two molecules.

It is necessary to explain the stereoselective, non-bonded and adsorbed sites of solute and solvent adsorptions on the crystal interface from the molecular level. Actually, all peripheral atoms of FOX-7 are involved in the intermolecular H-Bonds. From the neighbor contacting populations, Ma *et al*. confirmed that the O···H and N···H hydrogen bonds contribute 60% of the intermolecular forces in the crystal stacking of FOX-7^38^. Therefore, it is helpful to clearly explain the bonding behavior of the crystal faces to understand the anisotropy of the crystal face. PBC theory has proven to be an effective way to describe the most general characteristics of crystal morphologies. The growth of these faces needs to overcome potential barrier for the formation of two-dimensional nuclei. According to Hartman and Perdok, a flat face (*F*-face) must include two or more PBCs^8,9^. Bennema and Gilman assumed that the growth rate of the crystal face is proportional to the adsorption energy, the vertical direction of the crystal face has the least number of strong bonds, and the crystal surface is more exposed^1^. The PBC network diagrams of the important crystal faces of FOX-7 are shown in the Fig. 2. We summarize the distribution of the directional bond of the FOX-7 crystal in the longitudinal direction of the crystal face. It is found that the two largest bond energies on (101) are −4.07 and −5.08 kcal/mol. Those on (10$\bar{1}$) face are −3.96 and −1.56 kcal/mol, thus (10$\bar{1}$) grows more slowly than (101). The bond energies of the strongest two bond chains are −4.07 and −6.16 kcal/mol on (001) face. Those of (011) face are also −4.07 and −6.16 kcal/mol, which has a higher density of the bond network than the (001) face. The (110) and (11$\bar{1}$) faces contain more and denser bonds, especially the former, thus they grow much faster than other faces.

### FOX-7 morphologies of temperature controlled

The crystal growth undergoes three regions from the solution phase to the crystal phase, that is, a liquid phase region, an interface phase region and a crystal phase region^39–42^. In the liquid phase, the solute and the solvent are uniformly mixed at the molecular level. The solute molecules in a solution are combined with each other by strong chemical bonds, such as hydrogen bonds (4–120 kJ/mol), π-π stacking (1–50 kJ/mol). In the transition phase region, the growth unit gradually approaches the crystal by diffusion. On the side close to the crystal phase region, the solute content in the phase region is gradually increased due to the strong bonding force of the crystal face. The newly formed chemical bond acts to adjust the internal structure properly, and the overall bonding mode is closer to the crystal phase. On the contrary, the phase region contains more solvent components on the side close to the liquid phase region. The interaction between the growth units is weaker, and is more loosely arranged in the solution. As the growth unit further approaches the growth surface of the crystal, the growth unit selectively combines to the crystal face in a particular direction, ultimately achieving a growth from the liquid phase to the interior of the crystal lattice and promoting crystal growth in a particular crystallographic direction. Due to the anisotropy of the crystal structure, the three-phase regions which play an important role in the crystallization process will have different crystallographic directions, and the single crystal thereby establishes an anisotropic growth morphology surrounded by different crystal faces^43^.

**Figure 3:**
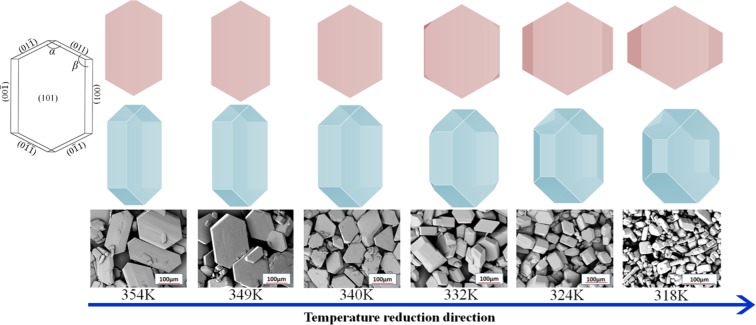
Simulated (upper) and experimental^37^ (lower) morphology of FOX-7 in DMSO/H_2_O = 2:1 at different temperatures. Red morphologies become blue ones after rotating 60°.

**Figure 4:**
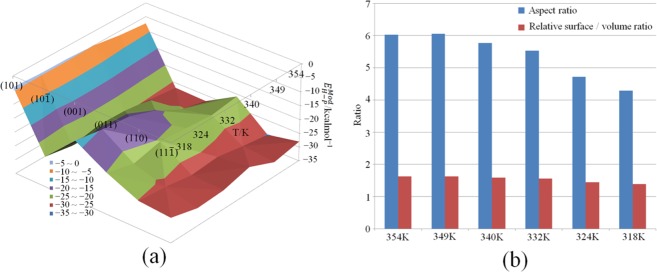
(**a**) Modified attachment energy and (**b**) aspect ratio of FOX-7 faces in DMSO/H_2_O = 2:1 and at different temperatures.

Temperature is a common experimental condition for controlling the anisotropic growth of organic materials. We used molecular dynamics to calculate the inhibition of different crystallographic directions of FOX-7, and to obtain a series of growth morphology evolutions. As shown in Fig. 3, the crystal shape changes regularly as the temperature changes in experiment. As the temperature is lowered, the size of a symmetrical face of the hexahedron becomes significantly smaller, the crystal shape changes from a slender hexagon to diamond, and the thickness of the crystal is remarkably increased. Our simulation obtained reproduces the above experimental results. Figure 4(a) shows the modified attachment energy of different crystal faces at different temperatures. The modified attachment energies were averaged over 5 times calculations. The attachment energies of (101), (10$\bar{1}$), (001), (011), (110) and (11$\bar{1}$) faces are −3.97 ± 0.01, −24.07 ± 0.08, −13.56 ± 0.04, −20.12 ± 0.06, −25.14 ± 0.04 and −26.07 ± 0.03 kcal/mol, respectively. The absolute error of adsorption energy is less than 0.1 kcal/mol. Table 2 lists the variation of the interplanar distance and surface area with temperature for the three main surfaces of (101), (001) and (011). Obviously, the modified attachment energy of (001) decreases rapidly, and that of (101) face slowly decreases with increasing temperature. However, the surface areas of other faces slowly rise. The roughness of the FOX-7 surface increases with increasing temperature. This facilitates the interaction of the solvent molecules with the crystal face. At the same time, the force of the solvent on the crystal face is reduced due to the temperature increasing. The simulated shapes are very consistent with the experiments at 318 K to 354 K. Figure 4(b) shows the aspect ratio and relative surface/volume ratio of FOX-7 face in DMSO/H_2_O = 2:1 at different temperatures. Obviously, the drop in temperature causes the aspect ratio of the FOX-7 crystal to decrease. In order to obtain a low aspect ratio, the FOX-7 should be crystallized at a lower temperature. However, the crystal shape in experiment at 318 K turns out to be a long strip with more crystal defects and more uneven particle sizes. The shape predicted at this temperature is different from the shape of the experiment. Liu believed that supersaturation is the driving force of crystallization and plays an important role in crystal growth rate of FOX-7^37^.

**Table 2:** Distance and area values for partial crystal faces of FOX-7 in DMSO/H_2_O = 2:1 and at different temperatures^a^.

T/K	surface	*d*_*hkl*_	${{\boldsymbol{R}}}_{{\boldsymbol{i}}}^{{\boldsymbol{Mod}}}{\boldsymbol{/}}{{\boldsymbol{R}}}_{{\boldsymbol{(}}101{\boldsymbol{)}}}^{{\boldsymbol{Mod}}}$	Area^b^
318	(101)	7.45	1.00	58.24
	(001)	27.19	3.65	4.00
	(011)	20.86	2.80	31.16
324	(101)	6.55	1.00	62.10
	(001)	24.69	3.77	5.88
	(011)	22.17	3.39	26.17
332	(101)	5.05	1.00	66.86
	(001)	19.58	3.87	11.44
	(011)	21.30	4.22	20.35
340	(101)	4.50	1.00	67.36
	(001)	16.19	3.60	14.09
	(011)	20.36	4.53	18.55
349	(101)	4.45	1.00	68.02
	(001)	15.18	3.41	15.93
	(011)	22.62	5.09	16.06
354	(101)	3.98	1.00	67.96
	(001)	13.56	3.41	15.90
	(011)	20.12	5.06	16.11

^a^All energies are in kcal mol^−1^, distances are in Å. ^b^Total facet area (%).

### Evolution path of FOX-7 morphology

**Figure 5:**
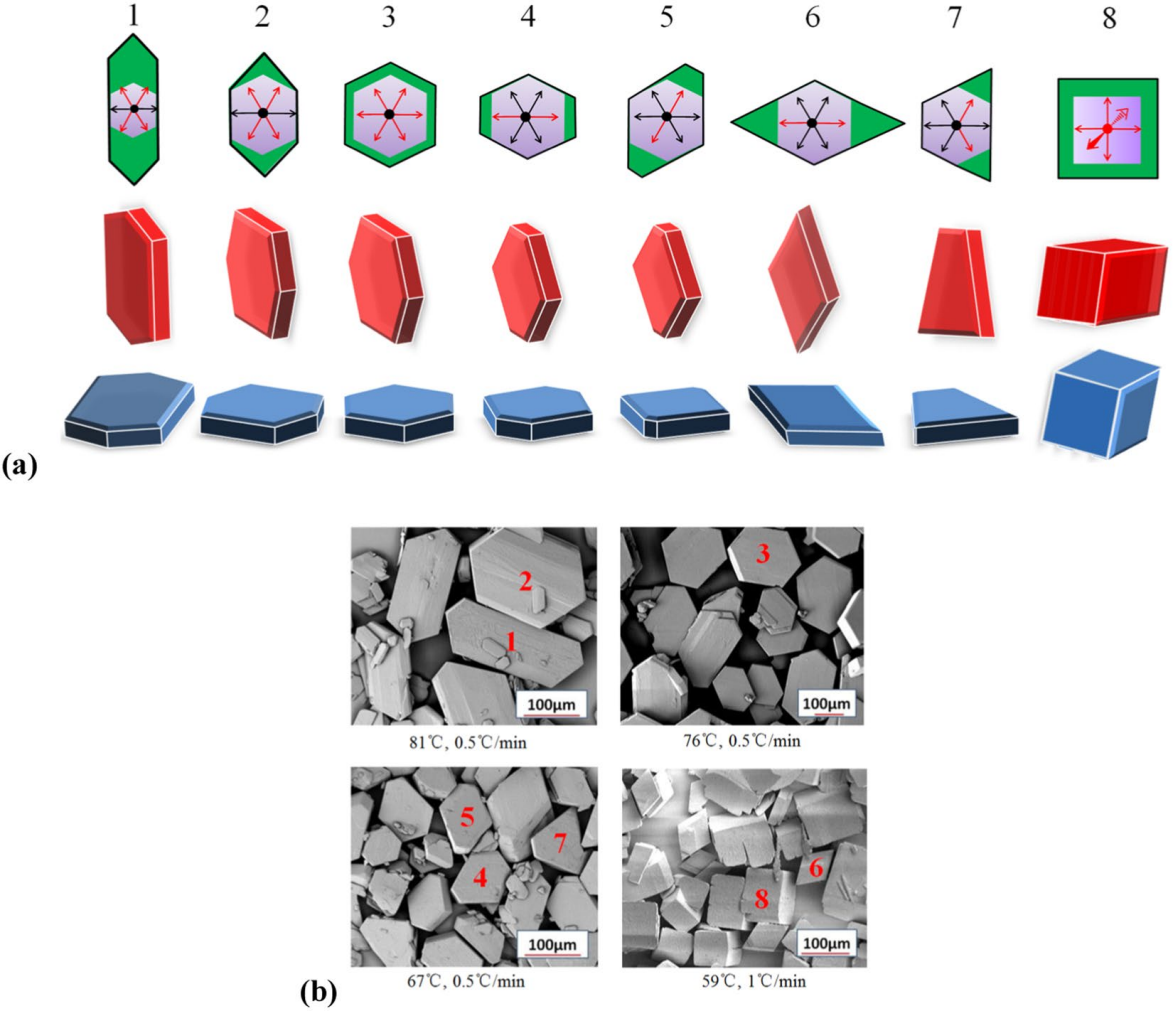
The evolution of predicted (**a**) and experimental (**b**) crystal shape, the numbers 1–8 represent the Route 1–8, respectively^37^. Red and blue are the same shapes viewed from different orientations. The red arrow represents the direction of fast growth, while the black represents the slow growth.

We have discussed that the FOX-7 shapes as a hexagon in DMSO/H_2_O. The adsorption energies of the six lateral faces, compared to the largest face (101), are slightly different. According to the Gibbs-Curie-Wulff theorem, any of the lateral faces, including (011), (001), (0$\bar{1}$1), (0$\bar{1}\bar{1}$), (00$\bar{1}$) and (10$\bar{1}$), have a chance to alter by different external environments, such as the fluctuations of temperature, pressure or flow field distribution of the solution^36^. In fact, the different shapes of crystalline product are found at different initial crystallization temperatures and cooling rates, and some examples are shown in Fig. 5. Routes 1 to 8 are growth directions indicated by the on-plane arrows (only two out-of-plane arrows in Route 8). For example, the symmetrical growth of (10$\bar{1}$), (011), (0$\bar{1}\bar{1}$) and (0$\bar{1}$1) faces result in the formation of elongated hexagonal crystals (Route 1). Symmetrical growth of the (001) and (00$\bar{1}$) faces produces diamond-shaped crystals with an internal angle of 59.78° (Route 6). The possibility of Route 7 explained the production of trapezoidal crystals. Here, we found that the vertical growth of FOX-7 also leads to the formation of quadrilateral crystals (Route 7) due to the symmetric growth of (101) and ($\bar{1}$0$\bar{1}$) faces.

## Conclusions

In summary, based on the H-P theorem, the anisotropic growth morphology of FOX-7 was calculated in vacuum. The H-P theorem of crystal growth reveals that the anisotropic morphologies of the materials depend on the surface bonding structure in different crystal directions. The modified H-P theorem predicts the hexagonal shape of FOX-7 at 354 K and explains the evolution of every crystal face at 318 to 354 K. This simulation directly demonstrates that the organic material has the property of temperature-controlled growth. The simulation results show that the (001) face is significantly reduced with the temperature decreases in DMSO/H_2_O = 2:1. At higher temperatures, the FOX-7 crystal appears as an elongated hexagon, and its crystal shape evolves to a diamond one with decreasing temperature. Our predicted all crystal morphologies of FOX-7 are in remarkable agreement with the experiments. At the same time, the crystal shapes of the FOX-7 undergo slight changes on different conditions. The tuning of the crystal shape helps to construct the crystalline materials with excellent properties and qualities. The simulation of crystal growth provided a new understanding of the crystal growth and morphology of FOX-7 crystals. It also has guidelines for obtaining the FOX-7 crystal with more excellent mechanical and safety properties.

## Methods

**Figure 6:**
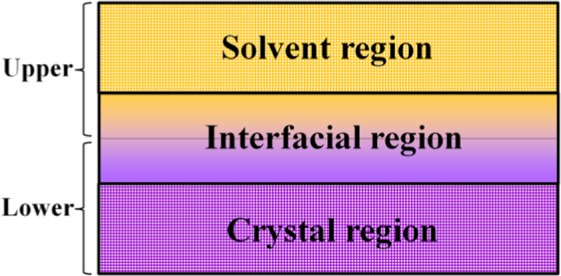
Schematic demonstration of the crystal growth process.

The H-P theorem of crystal growth was applied to the growth process of FOX-7, and the modified model was used to calculate the bonding behavior of anisotropic growth crystal faces in the growth environment, so as to realize the evolution trend of anisotropic growth morphology. Figure 6 shows a simplified model of the entire crystal growth process.

First, the interaction energy *E*_I_(S) between every crystal face and the solvent can be calculated as Eq. (1):

1 $${E}_{{\rm{I}}}({\rm{S}})={E}_{{\rm{T}}}({\rm{S}})-[{E}_{{\rm{U}}}({\rm{S}})+{E}_{{\rm{L}}}({\rm{S}})]$$

where ${E}_{{\rm{T}}}({\rm{S}})$, ${E}_{{\rm{U}}}({\rm{S}})$ and ${E}_{{\rm{L}}}({\rm{S}})$ are respectively the energy of total, upper and lower region in Fig. 6. Then, the modified attachment energy ${E}_{{\rm{H}}-{\rm{P}}}^{{\rm{Mod}}}$ (per unit area) that is affected by external conditions can be expressed as Eq. (2):

2 $${E}_{{\rm{H}}-{\rm{P}}}^{{\rm{Mod}}}={E}_{{\rm{H}}-{\rm{P}}}-\frac{{E}_{{\rm{I}}}({\rm{S}})}{R}$$

where ${E}_{{\rm{H}}-{\rm{P}}}$ is obtained by H-P theorem, *R* is the surface roughness. Thereby, the relative growth rate ${R}_{{\rm{hkl}}}^{{\rm{Mod}}}$ proportional to the ${E}_{{\rm{H}}-{\rm{P}}}^{{\rm{Mod}}}$ can be calculated as Eq. (3):

3 $${R}_{{\rm{hkl}}}^{{\rm{Mod}}}=\pm \,{\rm{k}}\times {E}_{{\rm{H}}-{\rm{P}}}^{{\rm{Mod}}}$$

**Figure 7:**
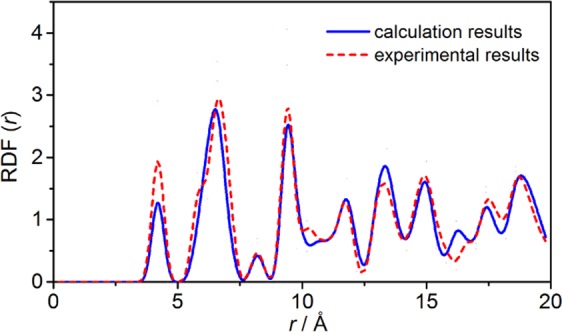
The radial distribution function of the optimized and experimental FOX-7 structure.

The theoretical simulations were performed with Material studio 5.5^44^, using COMPASS (Condensed-phase Optimized Molecular Potentials for Atomistic Simulation Studies) force field and Gasteiger charge^45^. The optimized structure of FOX-7 belongs to space group-*P2*_1_*/n*, the lattice parameters *a* = 6.88 Å, *b* = 6.56 Å, *c* = 11.41 Å, *α* = *γ* = 90° and *β* = 88.40°. The experimental structure of FOX-7 was obtained from the Cambridge structure database (CSD ref code: SEDTUQ) with *a* = 6.94 Å (0.83%), *b* = 6.57 Å (0.16%), *c* = 11.32 Å (0.87%), *α* = *γ* = 90° (0.00%) and *β* = 90.55° (2.38%)^46^. The temperature for the determination of crystal structure is 173 K. Figure 7 shows the comparison of the mass-center−mass-center radial distribution function (RDF) of all molecules in the optimized FOX-7 unit cell with experimental values. The RDF of the optimized FOX-7 unit cell agrees well with the experimental values. The calculated positions of main peak are 4.21, 6.64, 7.06, 9.45, 11.82 and 14.69, and the main peaks obtained from the experiment are 4.25, 6.64, 7.03, 9.48, 11.82 and 14.67. Therefore, the COMPASS force field can accurately predict the FOX-7 crystal structure. To simulate the crystal morphology in solvent, we first used the Morphology module to predict the crystal morphology to obtain some flat faces in vacuum. Then, the optimized unit cell is used as an initial configuration for slicing, expanding, and reconstructing into crystals as periodical supercell, and the crystal parameters (*a* and *b*) of this supercell are recorded to establish a solvent region. The two regions are docked to form a crystal-solvent bilayer configuration. It is worth noting that the crystal parameters (a and b) of the supercell are all larger than 45 Å for avoiding the effect of size, and the thickness of the solvent layer is twice that of the crystal layer. In order to avoid non-physical contact at the border of the simulated box, the bilayer model was relaxed for 50 ps NVT equilibrium at 298 K. Then, molecular dynamics simulation with isothermal-isobaric ensemble at a simulation time of 500 ps was performed for searching equilibrium state, and a time step of 1.0 fs is used. According to the experiment, five temperatures (318/324/332/340/354 K) and 1 bar were selected to simulate. The Andersen thermostat and Parrinello barostat was used to control the specific temperature and pressure^47^. The particle mesh Ewald summation is used for calculating both the electrostatic force and the van der Waals force^48^.
